# Clinical characteristics of angioid streaks in Japanese patients

**DOI:** 10.1007/s10384-026-01387-4

**Published:** 2026-07-03

**Authors:** Ruka Maruko, Ichiro Maruko, Nozomu Hashiya, Taiji Hasegawa

**Affiliations:** 1 Department of Ophthalmology, Tokyo Women’s Medical University, 8-1 Kawadacho, Shinjuku-ku, Tokyo, 162-8666 Japan; 2 Department of Ophthalmology, Uji Tokushukai Medical Center, 145 Ishibashi, Makishima-cho, Uji City, Kyoto, 611-0041 Japan; 3 Department of Ophthalmology and Visual Sciences, Kyoto University Graduate School of Medicine, 54 Shogoin Kawahara-cho, Sakyo-ku, Kyoto, 606-8507 Japan

**Keywords:** Angioid streaks, Pseudoxanthoma elasticum, Peau d’orange, Choroidal neovascularization, Japanese patients

## Abstract

**Purpose:**

To characterize fundus findings and the presence of pseudoxanthoma elasticum (PXE) in Japanese patients with angioid streaks (AS).

**Study design:**

Retrospective, single-center observational study.

**Methods:**

This retrospective study included 33 patients (66 eyes) diagnosed with AS who underwent color fundus photography, optical coherence tomography, and fundus autofluorescence. The presence of PXE, peau d’orange, choroidal neovascularization (CNV), macular atrophy, comet tail lesions, pattern dystrophy-like changes, subretinal drusenoid deposits (SDD), outer retinal tubulation (ORT), and optic nerve head drusen were evaluated.

**Results:**

PXE was diagnosed in 15 of 33 patients (45.5%), whereas peau d’orange was observed in 36 eyes (54.5%). There were no significant differences in the prevalence of peau d'orange (P=1.00) or comet tail lesions (P=0.70) between patients with and without clinical PXE diagnosis. CNV and macular atrophy were each present in 31 eyes (47.0%). Comet tail lesions, pattern dystrophy-like changes, SDD, and ORT were found in 17 (25.8%), 21 (31.8%), 6 (9.1%), and 13 (19.7%) eyes, respectively; optic nerve head drusen were not detected. Most pattern dystrophy-like changes (81.0%), SDD (83.3%), and ORT (92.3%) were associated with CNV.

**Conclusion:**

Japanese patients with AS showed a high frequency of CNV, which is often accompanied by macular atrophy and photoreceptor-related structural changes. Furthermore, the prevalence of peau d’orange was higher than of clinically diagnosed PXE, suggesting possible underdiagnosis of PXE in this cohort.

**Supplementary Information:**

The online version contains supplementary material available at https://doi.org/10.1007/s10384-026-01387-4.

## Introduction

Angioid streaks (AS) are a condition characterized by linear, crack‑like lesions radiating from the optic disc, resulting from calcification, degeneration, and rupture of elastic fibers in Bruch's membrane. They are frequently associated with choroidal neovascularization (CNV). Patients with concomitant pseudoxanthoma elasticum (PXE), characterized by systemic calcification and degeneration of elastic fibers primarily due to ABCC6 mutations, are known to be affected by Grönblad-Strandberg syndrome [1–3]. In PXE, besides CNV, a variety of fundus findings are known to occur, including orange peel-like punctate lesions (peau d'orange) [4], comet tail lesions, pattern dystrophy-like changes, optic nerve head drusen, subretinal drusenoid deposits (SDD), macular atrophy, and outer retinal tubulation (ORT) [5, 6].

However, AS is not a finding specific to PXE; it is reported to be associated with systemic diseases and local factors such as sickle cell disease, thalassemia, high myopia, and aging [7]. In routine clinical practice, even when AS is identified, comprehensive screening for systemic elastic fiber disorders including PXE is often not conducted, resulting in the potential existence of possibly underdiagnosed PXE cases that remain undetected.

Recently, intravitreal injections of anti-vascular endothelial growth factor (VEGF) drugs for CNV associated with AS have been approved in Japan, leading to renewed attention on AS and PXE [8]. This study evaluated PXE status and fundus findings in Japanese patients with AS.

## Methods

This was a retrospective study, and the procedures used conformed to the tenets of the Declaration of Helsinki. The Institutional Review Board of Tokyo Women's Medical University School of Medicine approved the procedures used. All examinations were performed as clinically indicated.

The study included 66 eyes of 33 patients (11 men, 22 women; mean age at diagnosis: 56.7 years) diagnosed with AS at the Department of Ophthalmology, Tokyo Women's Medical University. Fundus photography, optical coherence tomography (OCT), and fundus autofluorescence (FAF) were available for all patients. Visual acuity (converted to logarithm of the minimum angle of resolution, logMAR) and subfoveal choroidal thickness by OCT were also measured and evaluated. Subfoveal choroidal thickness was measured manually from the outer boundary of the retinal pigment epithelium (RPE) to the chorio-sclera interface using the built-in calipers of OCT software. No patients had a history of Paget disease, hemoglobinopathies, or other connective tissue disorders based on medical record review; systematic laboratory screening for hemoglobinopathies was not performed uniformly in all cases.

Angioid streaks were diagnosed when bilateral, irregular, dark red linear lesions radiating from the optic disc were confirmed on fundus photography and were consistent with linear breaks in Bruch's membrane. In some cases, confirmation was also obtained using near-infrared imaging or indocyanine green angiography.

This study investigated the presence of PXE, peau d'orange, CNV, macular atrophy, comet tail lesions, optic nerve head drusen, pattern dystrophy-like changes, SDD, and ORT in AS cases.

PXE was diagnosed according to the national diagnostic criteria for intractable diseases (*Shitei-Nanbyou*, No. 166) designated by the Ministry of Health, Labour and Welfare of Japan. The diagnostic criteria require: (1) characteristic skin lesions—yellowish papules arranged in a cobblestone pattern on flexural areas including the neck, axillae, and antecubital fossae; (2) ocular findings consistent with PXE, including angioid streaks and/or peau d’orange fundus appearance; and (3) histological confirmation by skin biopsy demonstrating calcification and fragmentation of elastic fibers in the mid-dermis. All 15 patients with PXE fulfilled these criteria and underwent dermatological examination and skin biopsy for histological confirmation. Genetic testing for the causative ABCC6 gene was not performed in this cohort.

Peau d'orange was defined by Smith and Gass [4] as a mottled fundus appearance resembling peau d'orange in PXE patients. In Japan, Shimizu [9, 10] reported this earlier as the *Nashiji* fundus. This term is derived from the textured surface of the Japanese pear fruit itself as well as the traditional Japanese lacquer technique *Nashiji maki-e*, both of which feature a surface with small irregularities.

Choroidal neovascularization was defined as the presence of exudative changes (serous retinal detachment, high-reflectivity lesions under the retina/RPE, hemorrhage, etc.) beneath or on top of the RPE along the AS, with new vascular lesions confirmed by fluorescein angiography or OCT angiography.

Macular atrophy was defined as a hypo-autofluorescent lesion on FAF. Since macular atrophy near the fovea can sometimes be difficult to visualize due to the presence of macular pigment, near-infrared FAF imaging was also utilized for confirmation in such cases.

​Comet tail lesions were defined as findings observed in the posterior pole to mid-periphery of the fundus, consisting of a small, yellowish-white, circular lesion with a faint, linear to triangular atrophic tail extending from the lesion toward the posterior pole. In this study, comet (comet-tail) lesions were documented using panoramic photography and/or ultra-widefield imaging including FAF.

​Optic nerve head drusen are observed as irregularly elevated, granular, hyperreflective lesions within the optic disc. They appear as hyperreflective nodules on OCT and show hyperfluorescence within the disc on FAF.

Pattern dystrophy-like changes refer to a group of diseases in adult-onset, macula-dominant hereditary retinal dystrophies in which yellowish-white deposits or pigment abnormalities located at the RPE level form distinct patterns such as butterfly, reticular, or patchy. All types of pattern dystrophy-like changes are reported to be observed in PXE [11, 12].

Subretinal drusenoid deposits differ from conventional drusen; these are subretinal, highly reflective deposits located superior to the RPE, near the photoreceptor ellipsoid zone. They are also called reticular pseudodrusen because they appear as a yellowish-white reticular pattern on color fundus photographs. In this study, lesions observed on OCT as drusen-like hyperreflective bands on the RPE were defined as SDD.

Outer retinal tubulation is a tubular (ring-shaped or rosette-shaped) structure with a continuous hyperreflective wall visible on OCT at the level of the outer plexiform to outer granular layer. ORT is observed in degenerative retinal diseases, such as advanced age-related macular degeneration and retinitis pigmentosa. It is thought that ORT lesions form through the rearrangement of the outer segments of cones and rods during the atrophic process. ORT is also frequently observed in PXE cases.

Statistical analyses were performed using SPSS software version 28.0 (IBM Corp.). Continuous variables are presented as mean ± standard deviation (SD). Differences between two groups were analyzed using the Mann–Whitney U test for continuous variables and Fisher's exact test for categorical variables. A P-value < 0.05 was considered statistically significant. To account for the inter-eye correlation between the right and left eyes of the same patient, generalized estimating equations (GEE; Gaussian family, exchangeable working correlation, robust sandwich standard error) and linear mixed models (LMM; random intercept, restricted maximum likelihood [REML]) were also used. Furthermore, 95% CIs and Cohen’s d were calculated to evaluate the effect size for key comparisons.

## Results

Of the 33 patients, four were lost to follow-up after the initial visit, while the remaining 29 were followed up. The mean follow-up period was 7.6 ± 6.0 years [range: 0.2-22.4 years]. The mean refractive error (spherical equivalent) was −2.67±3.26 D (range, −10.25 to +5.13 D). High myopia, defined as -6.0 D or less, was present in 10 eyes of 6 patients. Detailed clinical data and fundus findings for all individual patients are summarized in supplementary electronic table (Online Resource 1). The initial reason for consultation was: 22 patients (66.7%) were referred from local ophthalmology clinics due to fundus abnormalities, six (18.2%) were identified at routine health checkups, two (6.1%) self-referred due to decreased visual acuity, two (6.1%) were referred from a dermatologist for an unrelated condition, and one (3.0%) was referred from a cardiologist for an unrelated condition.

The mean logMAR best corrected visual acuity (BCVA) at diagnosis and at the final visit was 0.22±0.49 and 0.41±0.53, respectively; BCVA significantly decreased during follow-up (P<0.01). The mean subfoveal choroidal thickness also significantly decreased from 169±95 µm at baseline to 126±74 µm at the final visit (P<0.01).

**Fig. 1:**
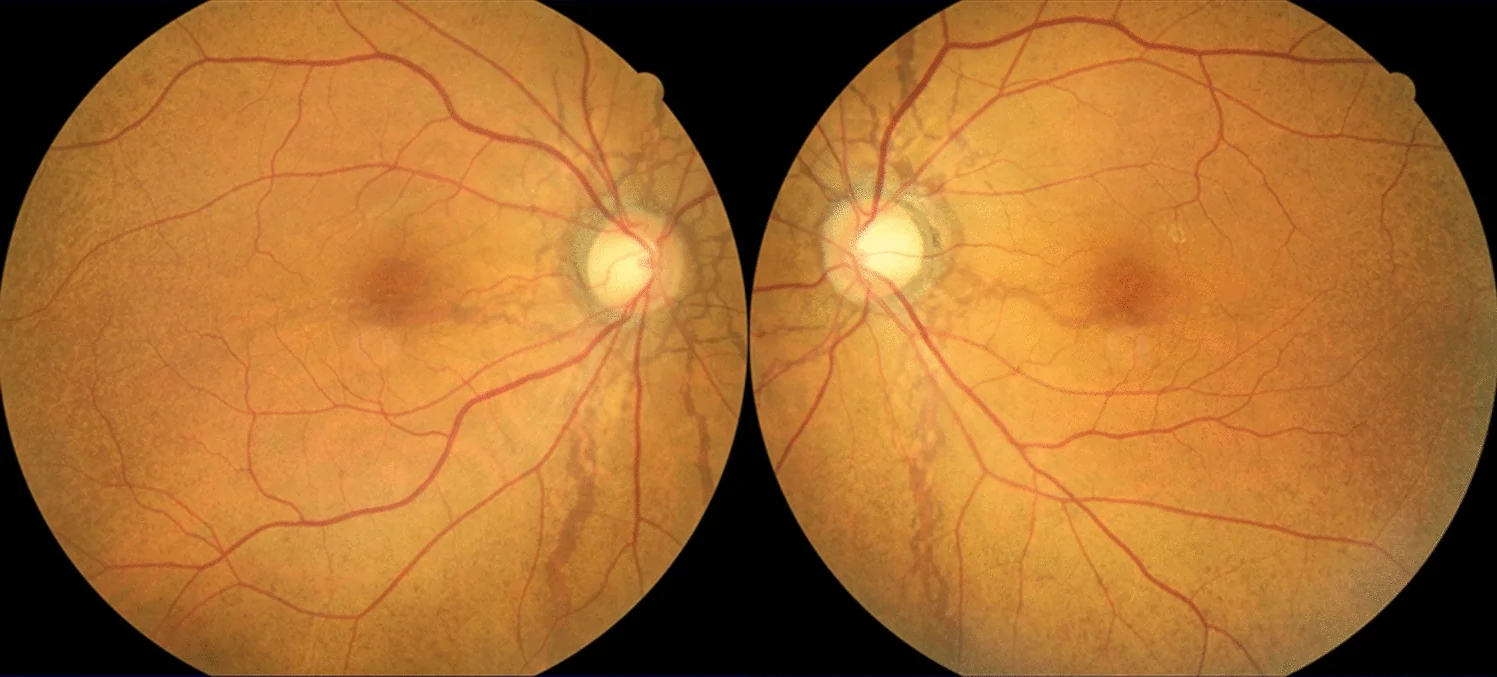
Angioid streaks and peau d’orange. Fundus photograph shows bilateral, irregular dark linear lesions radiating from the optic disc. Peau d'orange, a mottled fundus appearance, is visible in the temporal mid-periphery as subtle granular irregularities

**Fig. 2:**
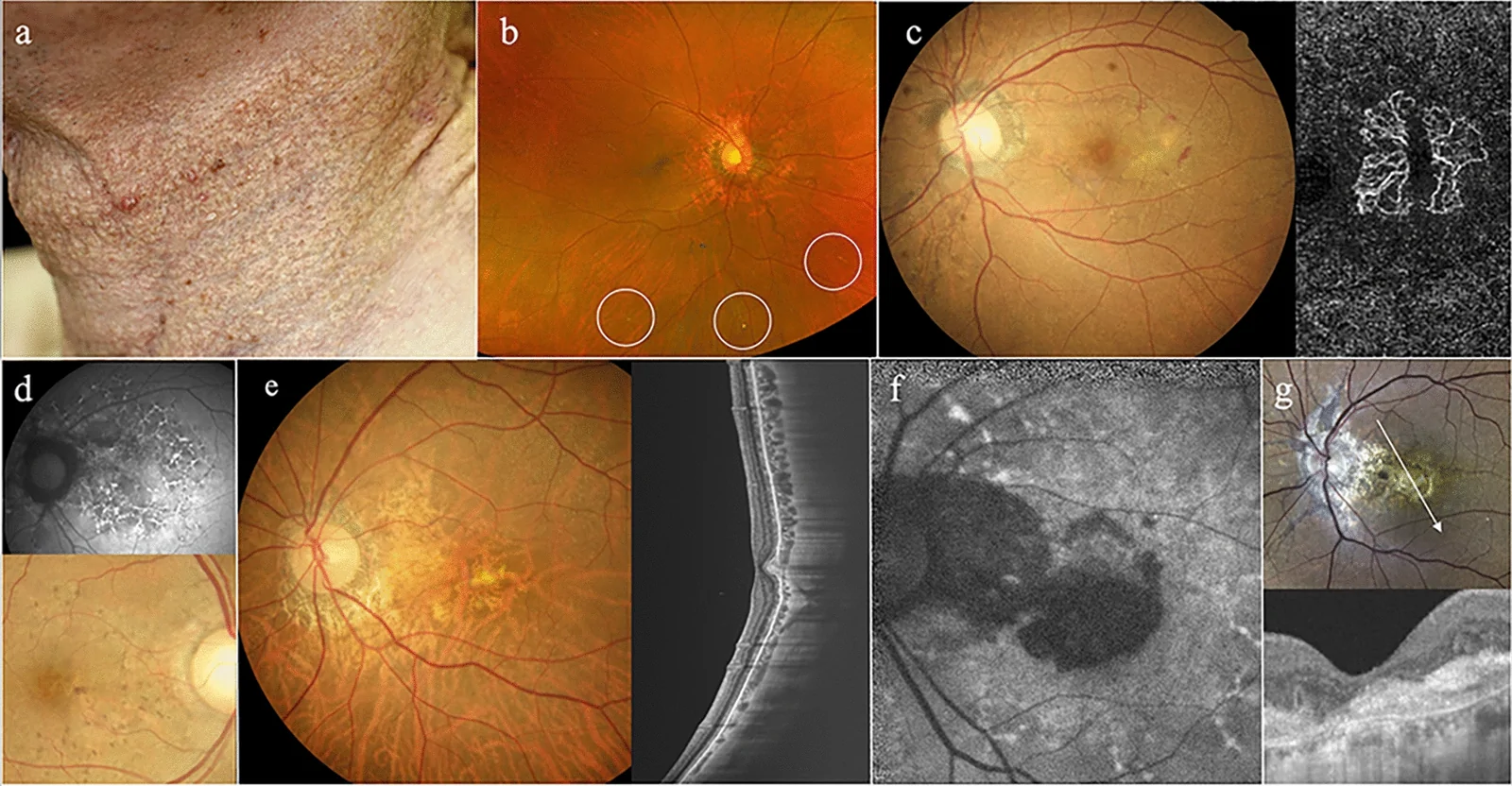
Fundus manifestations associated with angioid streaks. (**a**) Clinical manifestation of pseudoxanthoma elasticum (PXE) shows characteristic skin papules. (**b**) Fundus photograph shows comet tail lesions (circles), appearing as small nodules with tapering atrophic tails. (**c**) Choroidal neovascularization located temporal to the fovea, documented by fundus photography and optical coherence tomography angiography. (**d**) Pattern dystrophy-like changes show: the upper panel in fundus autofluoresein exhibits butterfly-shaped pigment dystrophy, characterized by radiating pigmentary pattern at the macula and the lower panel in fundus photograph shows fundus pulverulentus, characterized by fine, granular "salt-and-pepper" pigmentary changes. (**e**) Fundus photograph and corresponding vertical optical coherence tomography (OCT) scan shows subretinal drusenoid deposits as hyperreflective material located above the retinal pigment epithelium. (**f**) Extensive macular atrophy in a case with advanced angioid streaks. (**g**) Fundus photograph with a line scan (arrow) and the corresponding OCT section demonstrates outer retinal tubulation, visible as a circular structure with a continuous hyperreflective wall

Representative systemic and ocular manifestations evaluated in this study are shown in Figures 1 and 2. Figure 1 shows the characteristic peau d'orange and angioid streaks. Figure 2 displays a variety of findings, including the systemic skin lesions of PXE (Fig. 2a) and various fundus lesions such as comet tail lesions, pattern dystrophy-like changes, SDD, and ORT (Fig. 2b–g). Among the 33 AS patients at diagnosis, 15 (45.5%) had concomitant PXE. Peau d'orange was present in 36 eyes (54.5%), and CNV and macular atrophy were present in 31 eyes (47.0%) each. Comet tail lesions were present in 17 eyes (25.8%), pattern dystrophy-like changes in 21 eyes (31.8%), SDD in six eyes (9.1%), and ORT in 13 eyes (19.7%). Optic nerve head drusen were not detected. The prevalence of clinical characteristics in patients with AS is summarized in Table 1. At the patient level (at least one affected eye), peau d'orange was observed in 18 of 33 patients (54.5%), CNV in 17 patients (51.5%), macular atrophy in 16 patients (48.5%), comet tail lesions in 9 patients (27.3%), pattern dystrophy-like changes in 11 patients (33.3%), SDD in 4 patients (12.1%), and ORT in 7 patients (21.2%).

**Table 1:** Prevalence of clinical characteristics in the cases with angioid streaks

Parameter	Per eye (n = 66)		Per patient (n = 33)	
	n	%	n	%
PXE	15	45.45%	15	45.45%
peau d'orange	36	54.50%	18	54.50%
CNV	31	47.00%	17	51.50%
macular atrophy	31	47.00%	16	48.50%
comet tail	17	25.80%	9	27.30%
pattern dystrophy	21	31.80%	11	33.30%
SDD	6	9.10%	4	12.10%
ORT	13	19.70%	7	21.20%
ONHD	0	0.00%	0	0.00%

*PXE* pseudoxanthoma elasticum, *CNV* choroidal neovascularization, *SDD* subretinal drusenoid deposits, *ORT* outer retinal tubulation, *ONHD* optic nerve head drusen. For PXE, both per-eye and per-patient percentages represent the patient-level proportion. Ocular findings are reported both per eye (out of 66 eyes) and per patient (at least one affected eye, out of 33 patients).

To further investigate the relationship between systemic diagnosis and ocular findings, we compared the prevalence of characteristic fundus lesions between patients with and without a clinical diagnosis of PXE. Among the 15 patients with PXE, peau d’orange and comet tail lesions were present in 8 (53.3%) and 5 (33.3%) patients, respectively. Notably, among the 18 patients without a systemic PXE diagnosis, peau d’orange was identified in 10 patients (55.6%) and comet tail lesions in 4 patients (22.2%). There were no significant differences in the prevalence of peau d’orange (P=1.00) or comet tail lesions (P=0.70) between the two groups. These results suggest that typical fundus findings often occur regardless of a definitive systemic diagnosis.

Of the 17 patients with CNV (31 eyes), 14 cases (28 eyes) were bilateral. Two patients (three eyes) developed CNV during the follow-up period. Eyes with CNV (n = 31) exhibited significantly worse logMAR BCVA at diagnosis (0.52 ± 0.57; 95% confidence interval [CI], 0.32–0.72) compared to those without CNV (-0.06 ± 0.07; 95% CI, -0.08– -0.04, P < 0.01; Table 2). Similarly, subfoveal choroidal thickness was significantly lower in eyes with CNV (119 ± 58 µm; 95% CI, 98–140 µm) than in those without (213 ± 101 µm; 95% CI, 178–248 µm, P < 0.01; Table 2). The effect size for the difference in subfoveal choroidal thickness was large (Cohen’s d ≈ 1.12). These differences in LogMAR VA and subfoveal choroidal thickness remained highly significant after adjusting for inter-eye correlation using GEE and LMM (both P < 0.01).

**Table 2:** Visual acuity and subfoveal choroidal thickness between eyes with and without choroidal neovascularization.

Parameter	Eyes with CNV (n=31)	Eyes without CNV (n=35)	*P*-value
LogMAR VA, mean ± SD	0.52±0.57	−0.06±0.07	<0.01*
*95% CI*	*0.32–0.72*	*−0.08– −0.04*	
Mean SCT (µm), mean ± SD	119±58	213±101	<0.01*
*95% CI*	*98–140*	*178–248*	
High Myopia (n, %)	3 (9.7%)	7 (20.0%)	0.31†

logMAR, logarithm of the minimum angle of resolution; *VA* visual acuity, *SCT* subfoveal choroidal thickness, *SD* standard deviation, *CI* confidence interval, *CNV* choroidal neovascularization.

^*^Mann–Whitney U test

†Fisher’s exact test. Statistical significance for LogMAR VA and SCT was confirmed using GEE and LMM to account for inter-eye correlation (both P < 0.01). Cohen’s d for the difference in SCT was 1.12, indicating a large effect size.

**Fig. 3:**
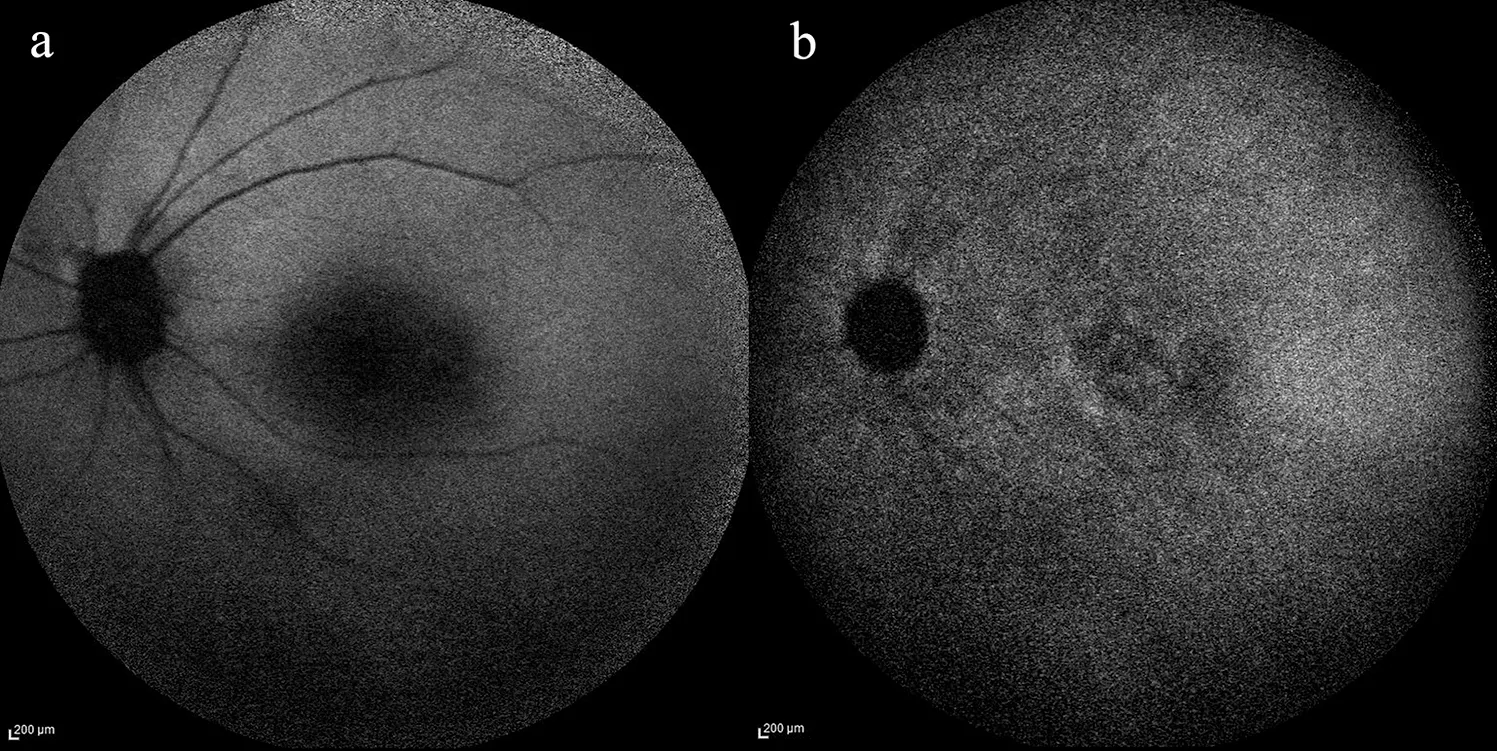
Macular atrophy unrelated to choroidal neovascularization. (**a**) Fundus autofluorescence image. The macular atrophy is difficult to identify due to macular pigment. (**b**) Near-infrared autofluorescence image. The area of macular atrophy is visualized as a distinct hypo-autofluorescent region

Furthermore, of the 16 cases (31 eyes) with macular atrophy, 15 cases (29 eyes) were associated with CNV. A representative case of non‑CNV‑related atrophy, confirmed using near-infrared FAF imaging, is shown in Figure 3. Most pattern dystrophy-like changes (81.0%), SDD (83.3%), and ORT (92.3%) were also associated with the presence of CNV.

Finally, a sub-analysis regarding refractive error showed that the mean subfoveal choroidal thickness was 146±82 µm in highly myopic eyes and 173±98 µm in non-highly myopic eyes, with no significant difference between the groups (P=0.38, Table 3).

**Table 3.** Subfoveal choroidal thickness in highly myopic versus non-highly myopic eyes.

Group	Number of eyes	Mean SCT (μm)	*P*-value*
High Myopia (SE ≤−6.0 D)	10	146±82	0.38
Non-High Myopia (SE >−6.0 D)	56	173±98	

SE, spherical equivalent. Data are presented as mean ± standard deviation. *Mann–Whitney U test.

*SCT* subfoveal choroidal thickness

## Discussion

The PXE diagnosis rate in AS patients was 45.5%, while peau d'orange was observed in 54.5% of eyes. This finding suggests the presence of undiagnosed PXE cases. This study found CNV or macular atrophy in approximately half of the eyes. Additionally, pattern dystrophy-like changes, SDD, and ORT, associated with photoreceptor damage, were observed in a significant number of cases, many of which were associated with CNV. These results suggest that, when AS is confirmed, a detailed fundus evaluation, including CNV, macular atrophy, and photoreceptor-related findings, is crucial in addition to a systemic evaluation for PXE.

Peau d'orange is considered a relatively specific early finding in PXE. Reports indicate that it appears early in many PXE patients in Western cohorts. However, the difference in frequency between PXE and peau d'orange observed in this study suggests the possible underdiagnosis of PXE, particularly in patients with mild or absent skin manifestations. Racial differences exist in PXE; the Japanese population shows different genetic variants compared to Western populations [13]. This may influence the frequency of cases presenting only with AS and peau d'orange without a PXE diagnosis. In our study, there was no significant difference in the frequency of peau d’orange between patients with and without PXE. Interestingly, 50% of patients without a PXE diagnosis still showed peau d’orange. Furthermore, comet tail lesions, another characteristic sign of PXE, were also observed in 25% of patients without a clinical PXE diagnosis. These findings raise the possibility of underdiagnosed PXE in Japanese patients and suggest that ocular signs may appear earlier or more clearly than skin symptoms. However, as comprehensive genetic testing and systematic exclusion of other systemic causes were not performed, this interpretation requires cautious consideration.​

Regarding the epidemiology of AS and PXE, Wada et al. [14] identified 6,598 AS cases and 1,020 PXE cases using Japan's national database. They report incidence rates of 0.52 and 0.08 per 100,000 person-years for AS and PXE, respectively. These rates indicate that, although AS and PXE are rare, their occurrence in the Japanese population may be more frequent than generally expected, consistent with the estimated prevalence in Western countries (PXE: 1/25,000–1/100,000) [5]. Although this study was a single-center cohort, the finding that the frequency of peau d'orange was higher than the PXE diagnosis rate supports the existence of cases with AS findings but without a PXE diagnosis, as shown in epidemiological studies.

​In the current study, CNV and macular atrophy were found in approximately half of the eyes, with most atrophy associated with CNV. In recent years, advances in imaging diagnostics, including OCT angiography, demonstrate that type 1 CNV is highly prevalent in AS-related CNV [15, 16]. The high number of bilateral CNV cases in this study further emphasizes the importance of early detection and follow-up of potential type 1 CNV in the fellow eye. In addition to these clinical features, the present study shows that subfoveal choroidal thickness was significantly reduced in eyes with CNV compared with those without CNV. Ellabban et al. [17] similarly report that the choroid in eyes with AS that had developed CNV was significantly thinner than both eyes with AS without CNV and normal control eyes. These findings together suggest that choroidal thinning may be closely related to the development or chronic course of CNV in eyes with AS, although it remains unclear whether choroidal thinning represents a predisposing factor for CNV or a consequence of longstanding disease activity. One potential concern is that the significant choroidal thinning observed in eyes with CNV could be attributed to a higher prevalence of high myopia in these cases. However, our sub-analysis revealed no significant difference in subfoveal choroidal thickness between highly myopic and non-highly myopic eyes. These findings suggest that the choroidal thinning associated with CNV is not a secondary effect of myopic changes, but rather a manifestation of the primary Bruch’s membrane and choroidal degeneration inherent to systemic elastic fiber disorders.

​As for macular atrophy, this study documented Bruch's membrane-related atrophy without CNV involvement, similar to previous reports, in addition to CNV‑associated atrophy [18–20]. One proposed mechanism suggests that severe calcification of Bruch's membrane and structural changes in the choroid disrupt the metabolic environment of the RPE and outer retina, leading to progressive atrophy regardless of CNV presence.

​In this study, pattern dystrophy-like changes, SDD, and ORT—all of which are associated with photoreceptor damage—were observed at a consistent frequency; the majority of these were related to CNV. Western PXE cohorts also report high rates of pattern dystrophy-like changes, subretinal deposits, and ORT around CNV lesions associated with AS [11, 12, 21, 22]. Furthermore, a multimodal retinal imaging analysis of PXE patients demonstrates that peau d'orange, comet tail lesions, RPE changes, CNV, and atrophy coexist in a layered distribution within the same case [23]. The results of this study suggest that similar morphological changes occur in Japanese AS cases.

​Interestingly, optic nerve head drusen were not detected in this cohort. These drusen are well documented in Western populations and may coexist with AS or PXE. However, a report indicates that optic nerve head drusen are less frequent in the general Japanese population than in Western populations, suggesting possible racial differences [24]. Future studies are needed to clarify the true frequency of optic nerve head drusen in the Japanese population.

This study has several limitations. First, as a single-center retrospective study with a limited number of cases, these results may not represent all Japanese AS patients. Second, as this is a university hospital-based cohort, the majority of patients were referred from local ophthalmology clinics with pre-identified fundus abnormalities, rather than through systematic population-based screening. Cases with visual impairment or CNV are therefore more likely to be represented, and the reported frequencies of CNV and macular atrophy may be higher than the true prevalence in all Japanese patients with AS. Third, although all 15 PXE patients underwent histological confirmation by skin biopsy according to the national *Shitei-Nanbyou* diagnostic criteria, ABCC6 genetic testing was not performed in any case. Given these limitations, future multicenter prospective studies combined with genetic analysis are necessary to verify the reproducibility and generalizability of the findings presented in this study.

AS is a condition in which Bruch's membrane elastic fibers degenerate and calcify due to congenital or acquired factors, resulting in linear breaks. On this common pathological background, the presence or absence of PXE, genetic background, racial differences, choroidal circulation, and environmental factors are thought to interact to produce diverse fundus phenotypes. The current study identified the frequency of CNV, macular atrophy, and photoreceptor‑related findings. This study also clarifies the discrepancy between the systemic PXE diagnoses and characteristic fundus findings in Japanese AS patients and provided valuable information about racial differences and phenotypic variation.

## Supplementary Information

Below is the link to the electronic supplementary material.

Supplementary file1 (DOCX 38 kb)

## References

[1] Grönblad E. Angioid streaks pseudoxanthoma elasticum: Vorläufige Mitteilung. Acta Ophthal. 1929;7:329.

[2] Strandberg J. Pseudoxanthoma elasticum. Z Haut Geschlechtskr. 1929;31:689 (**(in German)**).

[3] Germain DP. Pseudoxanthoma elasticum. Orphanet J Rare Dis. 2017;12:85. 10.1186/s13023-017-0639-8 PMC5424392 28486967

[4] Smith JL, Gass JD, Justice J Jr. fluorescein fundus photography of angioid streaks. Br J Ophthalmol. 1964;48:517–21. 10.1136/bjo.48.10.517 PMC506009 14221775

[5] Gliem M, Zaeytijd JD, Finger RP, Holz FG, Leroy BP, Charbel IP. An update on the ocular phenotype in patients with pseudoxanthoma elasticum. Front Genet. 2013;4:14. 10.3389/fgene.2013.00014 PMC3617449 23577018

[6] Pfau K, Lengyel I, Ossewaarde-van Norel J, van Leeuwen R, Risseeuw S, Leftheriotis G, et al. Pseudoxanthoma elasticum - Genetics, pathophysiology, and clinical presentation. Prog Retin Eye Res. 2024;102:101274. 10.1016/j.preteyeres.2024.101274 PMC12004504 38815804

[7] Nadelmann JB, Li Y, McGeehan B, Yu Y, VanderBeek BL. Systemic disease associations with angioid streaks in a large healthcare claims database. Eye (Lond). 2023;37:1596–601. 10.1038/s41433-022-02189-x PMC10220014 35915234

[8] Chugai Pharmaceutical Co., Ltd. Chugai obtains first regulatory approval in Japan for Vabysmo for additional indication of angioid streaks, a cause of vision loss. May 19, 2025. https://www.chugai-pharm.co.jp/english/news/detail/20250519153001_1157.htmlAccessed Jan 9, 2026.

[9] Shimizu K. Nashiji fundus in pseudoxanthoma elasticum. Jpn Rev Clini Ophthalmol. 1960;54:503–9 (**in Japanese**).

[10] Shimizu K. Mottled fundus in association with pseudoxanthoma elasticum. Jpn J Ophthalmol. 1961;5:1–13.

[11] Finger RP, CharbelIssa P, Ladewig MS, Scholl HPN, Holz FG, Schmitz-Valckenberg S. Fundus autofluorescence in pseudoxanthoma elasticum. Retina. 2009;29:1496–505. 10.1097/IAE.0b013e3181aade47 19823106

[12] Agarwal A, Patel P, Adkins T, Donald J, Gass JDM. Spectrum of pattern dystrophy in pseudoxanthoma elasticum. Arch Ophthalmol. 2005;123:923–8. 10.1001/archopht.123.7.923 16009832

[13] Katagiri S, Negishi Y, Mizobuchi K, Urashima M, Nakano T, Hayashi T. ABCC6 gene analysis in 20 Japanese patients with angioid streaks revealing four frequent and two novel variants and pseudodominant inheritance. J Ophthalmol. 2017;2017:1079687. 10.1155/2017/1079687 PMC5585540 28912966

[14] Wada S, Miyake M, Kido A, Kamei T, Hiragi S, Ikeda HO, et al. Epidemiology of angioid streaks and pseudoxanthoma elasticum (2011–2020): a nationwide population-based cohort study. Ophthalmol Sci. 2023;4:100370. 10.1016/j.xops.2023.100370 PMC10587625 37868801

[15] Marques JP, Bernardes J, Geada S, Soares M, Teixeira D, Farinha C, et al. Non-exudative macular neovascularization in pseudoxanthoma elasticum. Graefes Arch Clin Exp Ophthalmol. 2021;259:873–82. 10.1007/s00417-020-04979-z 33074374

[16] Cicinelli MV, Ramtohul P, Bianco L, Introini U, Bandello F, Freund KB, et al. Prevalence, features, and outcomes of type 1 neovascularization in eyes with angioid streaks. Ophthalmol Retina. 2025;9:166–79. 10.1016/j.oret.2024.08.002 39127109

[17] Ellabban AA, Tsujikawa A, Matsumoto A, Ogino K, Hangai M, Ooto S, et al. Macular choroidal thickness and volume in eyes with angioid streaks measured by swept source optical coherence tomography. Am J Ophthalmol. 2012;153:1133-43.e1. 10.1016/j.ajo.2011.12.013 22397956

[18] Risseeuw S, Ossewaarde-van Norel J, van Buchem C, Spiering W, Imhof SM, van Leeuwen R. The Extent of angioid streaks correlates with macular degeneration in pseudoxanthoma elasticum. Am J Ophthalmol. 2020;220:82–90. 10.1016/j.ajo.2020.07.022 32702361

[19] Sawa M, Gomi F, Tsujikawa M, Tano Y, McAllister IL, Mitchell P. Fundus autofluorescence in patients with pseudoxanthoma elasticum. Ophthalmology. 2006;113:814–20. 10.1016/j.ophtha.2006.01.037 16650677

[20] Gliem M, Müller PL, Birtel J, Hendig D, Holz FG, Charbel IP. Frequency, phenotypic characteristics and progression of atrophy associated with a diseased Bruch’s membrane in pseudoxanthoma elasticum. Invest Ophthalmol Vis Sci. 2016;57:3323–30. 10.1167/iovs.16-19388 27367499

[21] Kumar V. Reticular pseudodrusen and thin choroid are associated with angioid streaks. Ophthalmic Surg Lasers Imaging Retina. 2018;49:402–8. 10.3928/23258160-20180601-04 29927467

[22] GiachettiFilho RG, Zacharias LC, Monteiro TV, Preti RC, Pimentel SG. Prevalence of outer retinal tubulation in eyes with choroidal neovascularization. International Journal of Retina and Vitreous. 2016;2:6. 10.1186/s40942-016-0029-8 PMC5088468 27847624

[23] Zweifel SA, Imamura Y, Freund KB, Spaide RF. Multimodal fundus imaging of pseudoxanthoma elasticum. Retina. 2011;31:482–91. 10.1097/IAE.0b013e3181f056ce 20966826

[24] Komatsu H, Sano A, Yoneya S. Laser Photocoagulation for choroidal neovascularization developed in a patient with optic disc drusen and angioid streaks. Nippon Ganka Gakkai Zasshi. 2000;104:51–6 (**(in Japanese)**). 10659627

