# Supplementary material for: Clinical characteristics of angioid streaks in Japanese patients
Source: Jpn J Ophthalmol. 2026 Jul 3;70(5):1147–53. doi: 10.1007/s10384-026-01387-4 (PMC13616008; doi:10.1007/s10384-026-01387-4)
Supplement: Supplementary file 1 [file 10384_2026_1387_MOESM1_ESM.docx]

| **Online resource 1. Clinical characteristics and multimodal imaging findings of all patients with angioid streaks.** | | | | | | | | | | | | | | |  |  |  |  |  |  |  |  |  |  |  |  |  |  |
| --- | --- | --- | --- | --- | --- | --- | --- | --- | --- | --- | --- | --- | --- | --- | --- | --- | --- | --- | --- | --- | --- | --- | --- | --- | --- | --- | --- | --- |
|  |  |  |  |  |  |  |  |  |  |  |  |  |  |  |  |  |  |  |  |  |  |  |  |  |  |  |  |  |
| **No** | **Sex** | **Age (yr)** | **PXE** | **SE (D)** | | **logMAR VA** | | | | **SCT (µm)** | | | | **FU  (yr)** | **peau d'orange** | | **CNV** | | **macular atrophy** | | **comet tail** | | **pattern dystrophy** | | **SDD(RPD)** | | **ORT** | |
|  |  |  |  |  |  | **baseline** | | **final** | | **baseline** | | **final** | |  |  |  |  |  |  |  |  |  |  |  |  |  |  |  |
|  |  |  |  | **OD** | **OS** | **OD** | **OS** | **OD** | **OS** | **OD** | **OS** | **OD** | **OS** |  | **OD** | **OS** | **OD** | **OS** | **OD** | **OS** | **OD** | **OS** | **OD** | **OS** | **OD** | **OS** | **OD** | **OS** |
| **1** | F | 47.0 | 0 | -3.00 | -2.00 | -0.08 | -0.08 | -0.08 | -0.08 | 161 | 177 | 126 | 102 | 15.5 | 1 | 1 | 0 | 0 | 0 | 0 | 1 | 1 | 0 | 0 | 0 | 0 | 0 | 0 |
| **2** | M | 67.8 | 0 | -9.25 | 0.25 | 0.10 | -0.08 | 1.15 | -0.10 | 63 | 204 | 25 | 106 | 11.0 | 0 | 0 | 0 | 0 | 0 | 0 | 0 | 0 | 0 | 0 | 0 | 0 | 0 | 0 |
| **3** | M | 48.5 | 1 | -2.38 | -2.00 | 1.00 | -0.08 | 0.70 | 0.40 | 131 | 202 | 70 | 88 | 21.7 | 0 | 0 | 1 | 1 | 1 | 1 | 0 | 0 | 0 | 0 | 0 | 0 | 1 | 1 |
| **4** | F | 27.1 | 1 | -8.88 | -8.38 | -0.08 | 0.00 | - | - | 271 | 307 | - | - | - | 1 | 1 | 0 | 0 | 0 | 0 | 0 | 0 | 0 | 0 | 0 | 0 | 0 | 0 |
| **5** | M | 57.5 | 0 | -2.13 | -2.13 | 1.00 | -0.08 | 0.70 | 1.00 | 99 | 134 | 88 | 120 | 22.4 | 0 | 0 | 1 | 1 | 1 | 1 | 0 | 0 | 0 | 0 | 0 | 1 | 0 | 0 |
| **6** | F | 47.6 | 0 | -5.13 | -4.88 | -0.10 | -0.10 | -0.08 | -0.08 | 314 | 321 | 254 | 250 | 4.4 | 0 | 0 | 0 | 0 | 0 | 0 | 0 | 0 | 0 | 0 | 0 | 0 | 0 | 0 |
| **7** | M | 70.8 | 1 | 0.75 | 0.25 | 0.05 | 1.30 | 0.60 | 1.15 | 148 | 81 | 155 | 74 | 15.6 | 0 | 0 | 1 | 1 | 1 | 1 | 0 | 0 | 1 | 0 | 0 | 0 | 0 | 0 |
| **8** | F | 73.3 | 0 | -4.25 | -4.50 | -0.08 | 0.20 | -0.08 | -0.08 | 92 | 78 | 85 | 63 | 0.7 | 0 | 0 | 0 | 0 | 0 | 0 | 0 | 0 | 0 | 0 | 0 | 0 | 0 | 0 |
| **9** | F | 61.2 | 0 | -0.63 | -3.00 | -0.08 | -0.08 | 0.00 | 0.15 | 112 | 64 | 64 | 52 | 13.7 | 0 | 0 | 0 | 0 | 0 | 0 | 0 | 0 | 0 | 0 | 0 | 0 | 0 | 0 |
| **10** | M | 49.0 | 0 | -4.38 | -4.38 | 0.05 | 0.70 | 0.70 | 0.52 | 88 | 95 | 46 | 61 | 4.0 | 1 | 1 | 1 | 1 | 1 | 1 | 0 | 0 | 0 | 0 | 1 | 1 | 1 | 1 |
| **11** | F | 62.4 | 1 | 2.75 | 2.75 | -0.08 | 0.00 | 0.52 | 1.15 | 190 | 254 | 169 | 187 | 15.3 | 0 | 0 | 1 | 1 | 1 | 1 | 0 | 0 | 1 | 1 | 0 | 0 | 1 | 1 |
| **12** | F | 75.6 | 0 | -2.38 | 0.50 | -0.08 | 0.00 | 0.00 | 0.00 | 268 | 257 | 243 | 229 | 3.1 | 1 | 1 | 0 | 0 | 0 | 0 | 1 | 1 | 0 | 0 | 0 | 0 | 0 | 0 |
| **13** | M | 61.1 | 1 | -3.25 | -3.13 | -0.08 | 0.52 | 0.52 | 1.00 | 106 | 74 | 81 | 60 | 7.1 | 1 | 1 | 1 | 1 | 1 | 1 | 1 | 1 | 1 | 1 | 0 | 0 | 0 | 0 |
| **14** | F | 51.6 | 1 | -0.63 | 0.13 | -0.08 | -0.08 | -0.08 | -0.08 | 173 | 271 | 169 | 247 | 1.4 | 0 | 0 | 0 | 0 | 0 | 0 | 0 | 0 | 0 | 0 | 0 | 0 | 0 | 0 |
| **15** | F | 66.3 | 0 | 4.25 | 5.13 | -0.08 | 0.15 | -0.08 | 0.00 | 296 | 310 | 166 | 257 | 11.5 | 1 | 1 | 0 | 0 | 0 | 1 | 0 | 0 | 0 | 0 | 0 | 0 | 0 | 0 |
| **16** | M | 63.2 | 1 | -2.00 | -0.25 | 1.22 | 0.22 | 1.40 | 1.22 | 112 | 96 | 102 | 60 | 8.7 | 0 | 0 | 1 | 1 | 1 | 1 | 0 | 0 | 1 | 1 | 0 | 0 | 1 | 1 |
| **17** | F | 57.6 | 1 | -0.50 | -0.75 | -0.08 | -0.08 | 0.70 | 0.22 | 250 | 144 | 166 | 148 | 7.9 | 1 | 1 | 0* | 0* | 0 | 0 | 0 | 0 | 1 | 1 | 0 | 0 | 0 | 0 |
| **18** | F | 65.0 | 1 | -2.38 | -2.13 | -0.08 | 0.22 | -0.08 | 0.52 | 113 | 78 | 67 | 60 | 7.9 | 0 | 0 | 0 | 1 | 0 | 0 | 0 | 0 | 1 | 1 | 1 | 1 | 0 | 0 |
| **19** | M | 78.9 | 1 | -0.38 | 0.00 | -0.08 | -0.08 | 1.10 | 0.10 | 197 | 296 | 123 | 190 | 7.3 | 1 | 1 | 1 | 1 | 1 | 1 | 1 | 0 | 1 | 1 | 0 | 0 | 0 | 0 |
| **20** | M | 53.4 | 1 | -5.38 | -4.88 | 0.22 | 0.70 | 1.52 | 0.52 | 78 | 53 | 63 | 60 | 7.1 | 1 | 1 | 1 | 1 | 1 | 1 | 1 | 1 | 0 | 0 | 0 | 0 | 1 | 1 |
| **21** | M | 53.0 | 0 | -3.13 | -3.00 | -0.08 | -0.08 | -0.08 | -0.08 | 116 | 162 | 109 | 152 | 1.2 | 1 | 1 | 0 | 0 | 0 | 0 | 0 | 0 | 0 | 0 | 0 | 0 | 0 | 0 |
| **22** | F | 54.1 | 0 | -4.75 | -4.38 | -0.08 | -0.08 | 0.00 | 0.40 | 113 | 95 | 113 | 70 | 6.6 | 1 | 1 | 0* | 1 | 1 | 1 | 0 | 0 | 1 | 1 | 0 | 0 | 1 | 1 |
| **23** | F | 76.4 | 0 | 0.13 | -0.38 | 1.22 | 0.70 | 1.30 | 0.82 | 123 | 148 | 102 | 137 | 6.2 | 0 | 0 | 1 | 1 | 1 | 1 | 0 | 0 | 0 | 0 | 0 | 0 | 0 | 0 |
| **24** | F | 27.4 | 1 | -3.50 | -3.75 | -0.08 | -0.08 | -0.10 | -0.10 | 275 | 265 | 258 | 235 | 2.0 | 1 | 1 | 0 | 0 | 0 | 0 | 1 | 1 | 0 | 0 | 0 | 0 | 0 | 0 |
| **25** | F | 44.2 | 0 | -10.25 | -9.75 | -0.08 | -0.08 | -0.10 | -0.10 | 123 | 137 | 109 | 137 | 1.2 | 1 | 1 | 0 | 0 | 0 | 0 | 0 | 0 | 0 | 0 | 0 | 0 | 0 | 0 |
| **26** | F | 80.3 | 1 | 0.00 | -0.63 | 2.00 | 0.40 | 1.70 | 0.22 | 130 | 81 | 49 | 63 | 5.0 | 0 | 0 | 1 | 1 | 1 | 1 | 1 | 1 | 1 | 1 | 0 | 0 | 0 | 0 |
| **27** | F | 57.3 | 1 | -6.13 | -6.25 | -0.10 | -0.10 | -0.08 | -0.08 | 152 | 197 | 146 | 165 | 4.5 | 1 | 1 | 0 | 0 | 0 | 0 | 0 | 0 | 0 | 0 | 0 | 0 | 0 | 0 |
| **28** | F | 51.2 | 0 | -7.75 | -8.75 | 1.22 | 0.30 | - | - | 85 | 53 | - | - | - | 0 | 0 | 1 | 1 | 1 | 1 | 1 | 1 | 0 | 0 | 0 | 1 | 0 | 1 |
| **29** | M | 51.6 | 0 | -4.38 | -4.38 | 0.52 | 0.00 | 0.70 | 0.40 | 46 | 92 | 21 | 42 | 4.4 | 1 | 1 | 1 | 1 | 1 | 1 | 0 | 0 | 1 | 1 | 0 | 0 | 0 | 0 |
| **30** | F | 75.0 | 0 | -2.63 | -6.13 | 0.70 | 1.40 | 0.70 | 1.30 | 102 | 67 | 95 | 46 | 0.2 | 0 | 0 | 1 | 1 | 1 | 1 | 0 | 0 | 0 | 0 | 0 | 0 | 0 | 0 |
| **31** | F | 31.1 | 0 | -3.13 | -2.88 | -0.08 | -0.08 | 0.00 | 0.00 | 398 | 222 | 374 | 183 | 2.7 | 1 | 1 | 0 | 0 | 0 | 0 | 1 | 1 | 0 | 0 | 0 | 0 | 0 | 0 |
| **32** | F | 59.0 | 0 | 0.88 | 1.38 | 0.00 | 1.10 | - | - | 180 | 162 | - | - | - | 1 | 1 | 0 | 1 | 0 | 0 | 0 | 0 | 1 | 1 | 0 | 0 | 0 | 0 |
| **33** | F | 27.0 | 1 | -0.13 | 0.00 | -0.10 | -0.10 | - | - | 458 | 402 | - | - | - | 1 | 1 | 0 | 0 | 0 | 0 | 0 | 0 | 0 | 0 | 0 | 0 | 0 | 0 |
| **Mean** | **11:22** | **56.7 ± 14.6** | **15** | **-2.67 ± 3.26** | | **0.22** | | **0.41** | | **169 ± 95** | | **126 ± 74** | | **7.6 ± 6.0** | **18** | **18** | **14** | **17** | **15** | **16** | **9** | **8** | **11** | **10** | **2** | **4** | **6** | **7** |
|  |  |  |  |  |  |  |  |  |  |  |  |  |  |  |  |  |  |  |  |  |  |  |  |  |  |  |  |  |
| PXE, pseudoxanthoma elasticum; SE, spherical equivalent; logMAR, logarithm of the minimum angle of resolution; VA, visual acuity; SCT, subfoveal choroidal thickness; FU, follow-up; | | | | | | | | | | | | | | | | | | | | | | | | | | | |  |
| CNV, choroidal neovascularization; SDD, subretinal drusenoid deposits; RPD, reticular pseudodrusen; ORT, outer retinal tubulation. | | | | | | | | | | | | | | | | | | | | |  |  |  |  |  |  |  |  |
| *Asterisk indicates eyes that developed CNV during the follow-up period. | | | | | | | | | | |  |  |  |  |  |  |  |  |  |  |  |  |  |  |  |  |  |  |
